# Exercise and Neuropathic Pain: A General Overview of Preclinical and Clinical Research

**DOI:** 10.1186/s40798-021-00307-9

**Published:** 2021-03-22

**Authors:** Brianna N. Leitzelar, Kelli F. Koltyn

**Affiliations:** Department of Kinesiology, University of Wisconsin-Madison, 1300 University Ave., Madison, WI 53706 USA

**Keywords:** Pain management, Exercise training, Physical activity

## Abstract

Neuropathic pain is a disease of the somatosensory system that is characterized by tingling, burning, and/or shooting pain. Medication is often the primary treatment, but it can be costly, thus there is an interest in understanding alternative low-cost treatments such as exercise. The following review includes an overview of the preclinical and clinical literature examining the influence of exercise on neuropathic pain. Preclinical studies support the hypothesis that exercise reduces hyperalgesia and allodynia in animal models of neuropathic pain. In human research, observational studies suggest that those who are more physically active have lower risk of developing neuropathic pain compared to those who are less active. Exercise studies suggest aerobic exercise training (e.g., 16 weeks); a combination of aerobic and resistance exercise training (e.g., 10–12 weeks); or high-intensity interval training (e.g., 15 weeks) reduces aspects of neuropathic pain such as worst pain over the past month, pain over the past 24 h, pain scores, or pain interference. However, not all measures of pain improve following exercise training (e.g., current pain, heat pain threshold). Potential mechanisms and future directions are also discussed to aid in the goal of understanding the role of exercise in the management of neuropathic pain. Future research using standardized methods to further understanding of the dose of exercise needed to manage neuropathic pain is warranted.

## Key Points

- Animal studies demonstrate exercise training reduces pain behaviors (i.e., mechanical and thermal hyperalgesia and/or allodynia) in models of neuropathic pain.
- Human studies suggest moderate aerobic exercise training, combined aerobic and resistance exercise training, or high-intensity interval training reduces some, but not all, measures of neuropathic pain.
- Potential mechanisms of these effects include microglial activity, inflammatory markers, heat shock protein 72, neurotrophins, neurotransmitters, the opioid system, and the endocannabinoid system.

## Background

According to the International Association for the Study of Pain, neuropathic pain “is caused by a lesion or disease of the somatosensory system” and is typically characterized by spontaneous numbness, tingling/shooting, or burning pain. Neuropathic pain is difficult to treat, and few patients experience full relief. The complicated treatment and symptom burden of chronic neuropathic pain often comes with high economic burden. In fact, annual costs related to neuropathic pain (i.e., painful diabetic neuropathy; small fiber neuropathy; and neuropathic pain resulting from human immunodeficiency virus, HIV; spinal cord injury, SCI; chronic low back pain; or surgery/trauma) in 2011–2012 were estimated to be 27,259 US dollars per patient [1]. Thus, it is imperative to determine low cost and effective treatments to lessen this burden.

Neuropathic pain arises from damage or threat of damage to the somatosensory system that leads to maladaptive nociception [2–4]. Under normal conditions, nociception is activated when one encounters a damaging stimulus (i.e., touching a hot stove), under threat of damage, or upon tissue injury which activates protective responses (i.e., hand withdrawal from stove, healing response to injured site). Once a damaging stimulus is sensed, nociceptive neurons send signals from the site of injury (i.e., peripheral nerves), through the dorsal root ganglion (DRG), to the spinal cord (i.e., dorsal horn), which ultimately transports signals to the brain (i.e., ascending control). These ascending inputs are processed centrally, and descending outputs are carried down through the spinal cord and back to the dorsal horn to modulate pain signals (i.e., descending control) through excitatory and inhibitory neurons. Typically, ascending nociceptive signals cease once the stimulus (i.e., heat, tissue injury) is gone; however, some individuals develop chronic pain (i.e., maladaptive nociception). In the case of neuropathic pain, maladaptive signaling develops from damage to central or peripheral neurons.

The defining feature of neuropathic pain is the experience of pain in the absence of a stimulus (i.e., ectopic signaling) which is thought to result from nerve damage from injury (e.g., nerve injury), disease processes (e.g., diabetes; HIV), or external insults (e.g., chemotherapy treatment) that cause peripheral nerve sensitization (i.e., hyperexcitability of peripheral nerves) and/or central sensitization (i.e., hyperexcitability of nociceptive spinal neurons) [2–5]. As damaged nerves degenerate, areas of sensory loss form when the nerve is completely severed; however, any remaining fibers or fibers near the damaged site can send ectopic signals [2]. In the periphery, changes in ion channel sensitivity allow action potentials to be generated in response to low level inputs that would not trigger a pain signal under normal conditions [2–5]. Thus, frequent and spontaneous signals are sent from the periphery to the dorsal horn of the spinal cord. This increased peripheral input sensitizes spinal neurons, increasing ascending nociceptive input (i.e., central sensitization). In addition, central sensitization can be caused by maladaptive descending control as well as the loss of inhibitory interneurons in the DRG, which are controlled by systems such as serotonin, opioid, and endocannabinoid systems [6]. It is also thought that there is a strong neuroimmune response to nerve damage which contributes to peripheral and central sensitization [7]. Central and peripheral sensitization lead to hyperalgesia (i.e., exaggerated pain response to a painful stimulus) and/or allodynia (i.e., presence of pain response to an innocuous stimulus).

Current clinical practice guidelines focus primarily on pharmacological treatment [8–12]. Specifically, first-line treatments include anti-epileptics, tricyclic anti-depressants, topical treatments, and serotonin-norepinephrine reuptake inhibitors. Other pharmacological treatments include lidocaine or capsaicin patches, opioid agonists, and botulinum toxin A [2]. There are also guidelines on non-pharmacological interventions [10, 13, 14] which include neural blockade, surgical interventions, and spinal cord stimulation; however, these treatments are not effective for all types of peripheral neuropathies and may come with risks such as infection [2]. Other non-pharmacological treatments may include cranial and/or deep brain stimulation and intrathecal therapies that deliver drugs directly to affected neurons; however, the safety of these interventions has not been fully determined. Similarly, psychotherapy or complementary techniques such as acupuncture, Tai Chi Chuan, or exercise may be employed; however, the efficacy of such techniques is largely unknown.

Exercise may be a particularly important treatment option for patients with neuropathic pain, due to its wide array of established health benefits, such as reduced risk of chronic diseases including cardiovascular disease, type 2 diabetes, and cancer; reduced depression and anxiety; and improved sleep, cognition, bone health, and physical function [15]. However, before exercise can be recommended, researchers must establish its efficacy for patients who experience neuropathic pain. Therefore, the purpose of this review is to summarize the preclinical and clinical research examining the effects of exercise on neuropathic pain.

## Methodological Approach

A narrative review format was chosen to present a general overview of the literature to date. Since this is a burgeoning area of research, this overview will be helpful for researchers and clinicians who are interested in advancing the literature base. To do this, the authors searched databases (Academic Search Premier, Google Scholar, PubMed, and Web of Science) from their inception to the present for relevant literature and the last search was conducted on 2 June 2020. Relevant literature included observational and experimental studies examining the influence of physical activity or exercise on neuropathic pain. Search terms included terms related to neuropathy (e.g., diabetic peripheral neuropathy, DPN; chemotherapy-induced peripheral neuropathy, CIPN; HIV), physical activity (e.g., exercise, resistance training, aerobic exercise), and pain (e.g., neuropathic pain, hyperalgesia, allodynia). Preclinical studies were included if the animals studied were models of neuropathy, if animals underwent nociceptive testing, and at least one group underwent exercise training. Additionally, meta-analyses of preclinical literature were included. Observational and experimental human studies were included if the condition being studied related to neuropathic pain. In addition, observational evidence needed to measure physical activity to be included. Experimental evidence was included if at least one treatment arm was an exercise training program. Further, it is important to note that a distinction between neuropathy and neuropathic pain was made for this review and only studies where the authors could comment specifically on the impact of the exercise training on pain were included. In total, 44 preclinical studies were identified through the search criteria; 4 studies were excluded because the exercise intervention was combined with other treatments (*n* = 3) or there were no nociceptive tests included (*n* = 1). A total of 26 clinical studies were found; 14 studies were excluded because the assessments were not specific to neuropathic pain (*n* = 5), the impact of pain could not be isolated from neuropathy symptoms (*n* = 4), the study examined the acute impact of exercise (*n* = 1), or the intervention was not considered an exercise stimulus (*n* = 4).

## Preclinical Evidence

There is a plethora of preclinical evidence examining the influence of exercise on neuropathic nociception (i.e., responses to noxious stimuli), and this research is summarized in Table 1. Researchers use several animal models to understand the utility of exercise as a treatment for neuropathic pain by employing different behavioral tests to understand responses to noxious stimuli. Preclinical models include sciatic nerve injury (SNI), DPN, CIPN, complex regional pain syndrome (CRPS), and SCI. Common tests include applying mechanical (e.g., VonFrey filaments) and thermal (e.g., hot plates, acetone) stimuli and measuring the animals’ response. In general, aerobic exercise protocols reduce exaggerated responses (i.e., hypersensitivities) to these tests. In fact, recent meta-analyses concluded exercise training reduced nociceptive responses to mechanical and thermal tests compared to no exercise controls in animal models of peripheral nerve injury (i.e., SNI and DPN) [56] and SCI [57].

**Table 1:** Summary of preclinical evidence examining the impact of exercise training on neuropathic nociception

Study	Pain model	Exercise modality	Exercise program duration	Exercise Session (frequency and duration)	Exercise Intensity	Mechanism	Outcome
Kuphal et al. (2007) [16]	SNI	Swimming	Varied	90 min/day	Not indicated	NA	↓ Cold allodynia ↓ Thermal hyperalgesia
Cobianchi et al. (2010) [17]	SNI	Treadmill running	1 week 8 weeks	5 days/week 60 min/day	20–52 cm/s	Microglia	↓ Mechanical allodynia (1 week) ↔ Mechanical allodynia (8 weeks)
Bobinski et al. (2011) [18]	SNI	Treadmill running	2 weeks	5 days/week 30 min/day	10 m/min	Inflammation	↓ Mechanical hypersensitivity ↓ Cold hypersensitivity
Chen et al. (2012) [19]	SNI	Treadmill running Swimming	6 weeks 39 days	5 days/week 60 min/day 7 days/week 90 min	2–1.8 km/h	HSP72 and inflammation	↓ Heat sensitivity ↓ Mechanical allodynia
Shen and Cheng (2013) [20]	SNI	Swimming	4 weeks	5 days/week 60 min/day	Not indicated	NA	↓ Mechanical allodynia ↓ Thermal allodynia
Almeida et al. (2015) [21]	SNI	Swimming	5 weeks	5 days/week 10–50 min/day	Not indicated	Microglia, neurotrophins	↓ Mechanical hypersensitivity
Bobinski et al. (2015) [22]	SNI	Treadmill running	2 weeks	5 days/week 30 min/day	10 m/min	Serotonin	↓ Mechanical hypersensitivity
Kim et al. (2015) [23]	SNI	Treadmill running	4 weeks	5 days/week 30 min/day	8–22 m/min	Endogenous opioid system	↓ Mechanical allodynia ↓ Thermal allodynia
Lόpez- Àlvarez et al. (2015) [24]	SNI	Treadmill running	5 sessions 9 sessions	5 days/week 60 min/day	10–32 cm/s	Neurotrophins	↓ Mechanical hyperalgesia (5 and 9 session) ↓ Heat hyperalgesia (5 session)
Sheahan et al. (2015) [25]	SNI	Wheel running	2 weeks	Voluntary	Voluntary	NA	↔ Mechanical allodynia
Grace et al. (2016) [26]	SNI	Wheel running (prior to injury)	6 weeks	Voluntary	Voluntary	Microglia and inflammation	↓ Mechanical allodynia
Kami et al. (2016) [27]	SNI	Treadmill running	1 week	5 days/week 60 min/day	7 m/min	GABA	↓ Mechanical allodynia ↓ Heat hyperalgesia
Kami et al. (2016) [28]	SNI	Treadmill running	1 week	5 days/week 60 min/day	7 m/min	Microglia	↓ Mechanical allodynia ↓ Heat hyperalgesia
Wakaizumi et al. (2016) [29]	SNI	Treadmill running	2 weeks	5 days/week 60 min/day	6 m/min 12 m/min	Dopamine	↓ Tactile allodynia (both) ↓ Thermal hyperalgesia (both)
Safakhah et al. (2017) [30]	SNI	Treadmill running	3 weeks	5 days/week 30 min/day	16 m/min	Inflammation	↓ Mechanical allodynia ↓ Thermal allodynia
Tsai et al. (2017) [31]	SNI	Treadmill running	3 weeks	7 days/week 30 min/day	14–16 m/min @ 8% grade	Inflammation	↓ Mechanical allodynia ↓ Heat allodynia
Bobinski et al. (2018) [32]	SNI	Treadmill running	2 weeks	5 days/week 30 min/day	10 m/min	Inflammation, microglia, neurotrophins	↓ Mechanical hyperalgesia
Lόpez- Àlvarez et al. (2018) [33]	SNI	Treadmill running	12 days	60 min/day	10–32 cm/s	Adrenergic and serotonin systems	↓ Mechanical allodynia ↓ Thermal allodynia
Sumizono et al. (2018) [34]	SNI	Treadmill running	5 weeks	5 days/week 30 min/day 3 days/week 30 min/day	20 m/min	Microglia, neurotrophin, endogenous opioid system	↓ Mechanical sensitivity (both)
Hutchinson et al. (2004) [35]	SCI	Treadmill running Swim training	7 weeks	5 days/week 20–25 min/day	Not indicated	Neurotrophins	↓ Mechanical hyperalgesia (all) ↓ Mechanical allodynia (treadmill) ↓ Mechanical allodynia- transient (swim)
Stagg et al. (2011) [36]	SCI	Treadmill running	5 weeks	5 days/week 30 min/day	14–16 m/min @ 8% grade	Endogenous opioids	↓ Mechanical allodynia ↓ Heat allodynia
Detloff et al. (2014) [37]	SCI	Forced running wheel	6 weeks	5 days/week 20 min/day	5–14 m/min	Neurotrophins	↓ Tactile allodynia ↔ Thermal hyperalgesia
Ward et al. (2014) [38]	SCI	Body weight supported treadmill running	81 days	7 days/week 58 min/day	10–22 cm/s	Neurotrophins	↓ Mechanical allodynia
Dugan and Sagan (2015) [39]	SCI	Treadmill running	15 weeks	5 days/week 10–40 min/day	11–15 m/min @ 8° incline	NA	Prevention of heat hyperalgesia and cold allodynia ↓ Mechanical allodynia ↓ Heat hyperalgesia ↓ Cold allodynia
Detloff et al. (2016) [40]	SCI	Forced running wheel (delayed exercise)	5 weeks	5 days/week 20 min/day	6–14 m/min	NA	↔ Mechanical allodynia (animals with allodynia pre-exercise) ↑ Mechanical allodynia (animals without allodynia pre-exercise)
Nees et al. (2016) [41]	SCI	Treadmill	5 weeks	2×/day 15 min/session 5 days/week	Not specified	Fiber density	↓ Mechanical allodynia ↔ Heat hyperalgesia ↔ Loss of cold sensitivity (hypoalgesia)
Ward et al. (2016) [42]	SCI	Bodyweight supported treadmill training	6 weeks	6 days/week 30 min/day	22 cm/s	NA	↔ Mechanical allodynia
Gong et al. (2017) [43]	SCI (infant)	Wheel running	Unclear	7 days/week	Voluntary	Microglia, inflammation	NA
Chhaya et al. (2019) [44]	SCI	Forced running wheel	4 weeks	5 days/week 20 min/day	14 m/min	Macrophages	↔ Mechanical allodynia
Dugan et al. (2020) [45]	SCI	Treadmill running	10 weeks	5 days/week 10–40 min/day	10–15 m/min @ 8^0^ incline	Inflammation	Prevention of mechanical allodynia, heat hyperalgesia, and cold allodynia ↓ Mechanical allodynia ↓ Heat hyperalgesia ↓ Cold allodynia
Groover et al. (2013) [46]	High-fat diet (Pre-diabetes)	Voluntary wheel running	12 weeks	Voluntary	Voluntary	Neurotrohpins and epidermal innervation	↓ Mechanical allodynia ↔ Thermal allodynia
Rossi et al. (2011) [47]	DPN	Group swimming	8 weeks	7 days/week 10–60 min/day	Not indicated	NA	↓ Thermal hyperalgesia
Shankarappa et al. (2011) [48]	DPN	Treadmill running	10 weeks	5 days/week 60 min/day	18 m/min	Endogenous opioid system	↓ Mechanical allodynia
Chen et al. (2013) [49]	DPN	Treadmill Running	8 weeks	Daily	20–25 m/min	HSP72	↓ Mechanical hypersensitivity ↓ Heat hypersensitivity
Aghdam et al. (2018) [50]	DPN	Swimming	10 weeks	5 days/week 60 min/day	Not indicated	miR-96	↓ Thermal pain threshold
Golbar et al. (2018) [51]	DPN	Treadmill running	6 weeks	5 days/week 10–30 min/day	10–18 min/min	Motor proteins	↓ Mechanical allodynia ↓ Thermal hyperalgesia
Ma et al. (2018) [52]	DPN	Treadmill running	5 weeks	4 days/week 10 min/day	5–10 m/min @ 10% grade	Inflammation	↓ Mechanical hyperalgesia
Martins et al. (2013) [53]	CRPS Type I	Swimming	4 weeks	7 days/week 50 min/day	Not indicated	Endogenous opioid and adenosinergic systems	↓ Mechanical allodynia
Park et al. (2015) [54]	CIPN	Treadmill running	4 weeks	7 days/week 50 min/day	10 m/min	Epidermal innervation	Exercised mice did not develop thermal hypoalgesia.
Slivicki et al. (2019) [55]	CIPN	Voluntary wheel running	23–28 days	7 days/week	Voluntary	NA	Mice who exercised before and during Paclitaxel treatment did not develop mechanical or cold allodynia Mice who ran before Paclitaxel had delayed onset of mechanical and cold allodynia Mice who ran during Paclitaxel treatment had less cold allodynia.

Studies are grouped by pain model then organized chronologically. *CIPN* Chemotherapy-induced peripheral neuropathy, *CRPS* Complex regional pain syndrome, *DPN* Diabetic peripheral neuropathy, *SNI* Sciatic nerve injury, *SCI* Spinal cord injury, ↓ = lower NPP outcome in exercised mice compared to sedentary, ↑ = higher NPP outcome in exercised mice compared to sedentary, ↔ = no differences in NPP outcome between groups

A brief look at the wider literature supports the conclusions of the aforementioned meta-analyses. Specifically, 1–6 weeks of treadmill running [17–19, 22–24, 27–34] and 6–39 days of swim training [16, 19–21] reduced mechanical and thermal indices of nociception in SNI models of neuropathic pain. Further, there is consistent evidence that 7–14 weeks of treadmill running reduced mechanical indices of nociception in SCI models of neuropathic pain [38, 39, 45]. While there is less literature in DPN, CIPN, and CRPS models, in general, 4–10 weeks of treadmill running [48, 49, 51, 52, 54], 4–10 weeks of swim training [47, 50, 53], and 23–28 days of wheel running [55] ameliorated abnormal nociception in these models. Other durations of exercise training are less conclusive [17, 24, 25, 40–42, 44], suggesting there may be an optimal exercise duration for effective neuropathic pain management. Further, one study demonstrated wheel running induced mechanical allodynia when it started 2 weeks after nerve injury [40], which requires further investigation. Also, it is important to note that animal models are not fully representative of the human pain experience [58]; thus, it is important to understand if human research demonstrates similar benefits of exercise. Accordingly, the purpose of the following section is to provide an overview of the clinical research to date.

## Clinical Evidence

In the wider chronic pain (i.e., pain lasting more than 3 months or past normal tissue healing) literature, exercise is considered an important component of effective chronic pain management. In fact, treatment guidelines published by the Centers for Disease Control suggest including exercise as a part of the treatment plan for chronic pain that should be used before opioid-based pharmacological treatments [59]. Observational evidence suggests regular physical activity (i.e., 2–3 days per week of exercise) reduces the risk for chronic pain by 10–15% in those younger than 65 and by 20% in those 65 years or older [60]. Further, meta-analytic evidence suggests small to moderate reductions in pain in individuals with fibromyalgia [61, 62], moderate reductions in chronic low back pain [63], and small benefits for improving knee osteoarthritis [64, 65]. While there is consistent evidence that chronic exercise training is beneficial for reducing pain in chronic low back pain, fibromyalgia, and knee osteoarthritic samples, the clinical literature base for individuals with neuropathic pain is in its infancy.

### Observational Evidence

Observational evidence suggests physical activity is associated with lower pain in colorectal cancer survivors treated with chemotherapy [66] and individuals with type 2 diabetes [67, 68] (see Table 2). Specifically, colorectal cancer survivors treated with chemotherapy and who were active (i.e., reported 150 min of moderate-intensity physical activity per week) were less likely than those who were inactive to report pain and other symptoms of CIPN such as trouble standing/walking, weakness, cramps, and loss of strength in their hands [66]. In addition, when only participants who reported CIPN scores in the top 30% (i.e., severe CIPN) were included, active participants reported experiencing less pain, lower fatigue scores, and higher function compared to individuals who were inactive. In individuals with diabetes, the evidence demonstrates that physical inactivity is a risk factor for painful diabetic neuropathy [67, 68]. For example, this was demonstrated in a cohort of individuals with diabetes following myocardial infarction in which participants reported their leisure time physical activity [67]. The results indicated that, in combination with smaller waist circumference and the absence of peripheral arterial disease, participants who reported at least 1 h per week of physical activity were less likely to have neuropathic pain. Chiang et al. (2016) examined different levels of exercise: no exercise, inactive (i.e., less than 30 min per day of exercise), or active (i.e., 30 min or more per day of exercise) [68]. Their results indicated that compared to active individuals, the no exercise and inactive groups were at a higher risk of having neuropathic pain. The body of observational research suggests that being physically active reduces the risk of experiencing neuropathic pain. Even so, experimental studies are needed to understand the direct impact of exercise on neuropathic pain and to begin to understand the dose (i.e., frequency, intensity, type, and time) needed to manage neuropathic pain.

**Table 2:** Summary of clinical evidence examining the impact of exercise on neuropathic pain

Study	Diagnosis	Condition Groups	Exercise Duration	Exercise Session (frequency and duration)	Exercise Intensity	Outcome
Ziegler et al (2009)^a^ [67]	Diabetes and myocardial infarction	NA	NA	NA	NA	Individuals who reported at least 1 h a week of leisure time sports activities year-round were at a lower risk for having neuropathic pain than those who reported less sport activity.
Kluding et al. (2012) [69]	Diabetes and metabolic syndrome	Combined (AET and RT)	10 weeks	3–4 days/week 30–50 min *AET* 2–4 days/week *RT* 1–2 days/week 10–20 reps	Moderate (50–70% VO_2_max)	↔ Current pain ↔ Usual pain ↓ Worst pain over the past month ↑ Intraepidermal nerve fiber branching
Hamed and Raoof (2014) [70]	Diabetes and obesity	Aerobic	15 weeks	Moderate 3 days/week 50 min HIIT 3 days/week 20 min	Moderate (50–60% MHR) Vigorous (85–95% MHR)	↓ Pain for HIIT compared to moderate intensity
Yoo et al. (2015) [71]	Diabetes	Aerobic	16 weeks	3 days/week 30–50 min/day	Moderate (50–70% VO_2_ reserve)	↔ Pain intensity ↓ Pain interference
Chiang et al (2016)^a^ [68]	Diabetes	NA	NA	NA	NA	Participants who reported 30 min or more per day of exercise had lower odds of having neuropathic pain compared to those who reported less than 30 min per day of exercise and those who reported no exercise.
Nadi et al. (2017) [72]	Diabetes	Vitamin D only Vitamin D + combined (AET and RT)	12 weeks	3 days/week 60 min/day	Moderate (*AET* 50-70% MHR *RT* 50% 10-RM)	↓ Pain
Stubbs Jr. et al. (2019) [73]	Diabetes	Health education Aerobic Resistance Combined (AET and RT)	12 weeks	Health Education: 12 sessions Exercise: 3 days/week 30–45 min/day	Moderate (60–90% VO_2peak_)	↔ Vibration detection threshold (all programs) ↔ Cooling detection threshold (all programs) ↔ Heat pain threshold (all programs)
Cox et al. (2020) [74]	Diabetes	Usual care Combined (AET and RT) Combined HIIT (AET and RT)	8 weeks	Combined: 4 days/week 52.5 min/day Combined HIIT: 3 days/week 26 min/day	Moderate (55–69% HRpeak; fairly light to somewhat hard) Vigorous (85–95% HRpeak; very hard)	↓ Musculoskeletal pain (combined HIIT only) ↔ Neuropathy symptoms (all conditions) ↔ Cooling detection threshold (all conditions) ↔ Warm detection threshold (all conditions) ↔ Vibration detection threshold (all conditions) ↔ Mechanical detection threshold (all conditions) ↔ Vibration detection threshold (all conditions) ↔ Pressure pain threshold (all conditions)
Wonders et al. (2013) [75]	CIPN	Home-based combined (AET and RT)	10 weeks	*AET:* 2–5 days/week 10–30 min/day *RT:* 3 days/ week	Moderate (55–65% MHR)	↓ Pain
Dhawan et al. (2019) [76]	CIPN	Usual care Home-based (RT and balance)	10 weeks	*RT & Balance* 7 days/week 30 min/day	Not indicated	↓ Pain (RT and balance)
Mols et al. (2015)^a^ [66]	Colorectal cancer survivors	NA	NA	NA	NA	There were fewer people who reported aching/burning pain, trouble handling small objects, pain, trouble standing/walking, weakness, cramps, and loss of strength in their hands in individuals who had chemotherapy and met the national PA guidelines compared to individuals who had chemotherapy and did not meet the national PA guidelines.
Maharaj et al. (2018) [77]	HIV distal symmetrical polyneuropathy	Control: HIV education Aerobic Resistance	12 weeks	3 days/week 30 min/day	Low–moderate (*AET* 40–65% MHR *RT* 40–65% 1-RM)	↓ Pain intensity (AET; RT)

^a^indicates an observational study

Studies are grouped by diagnosis then organized chronologically. *AET* Aerobic training, *CIPN* Chemotherapy-induced peripheral neuropathy, *HIV* Human immunodeficiency virus, *HRpeak* Peak heart rate, *MHR* Maximal heart rate, *NA* Not applicable, *rep* Repetition, *RT* Resistance training, *RM* Repetition maximum test, ↓ = decrease in outcome, ↑ = increase in outcome, ↔ = no change in outcome

### Exercise Training and Neuropathic Pain in Adults

#### DPN

To date, the majority of exercise training studies have been conducted with participants diagnosed with diabetes or DPN [69–74]. Different exercise training programs have been examined including aerobic exercise training, combined aerobic and resistance exercise training, and high-intensity interval training (HIIT).

##### Single-Arm Studies

Two studies examined the influence of chronic exercise training using a single-arm study design [69, 71]. In one study, participants with DPN and metabolic syndrome reported significantly lower “worst pain over the past month,” reduced neuropathy symptoms (e.g., numbness, burning, tactile sensitivity, pain) and had significantly more intraepidermal nerve fiber branching following a 10-week progressive aerobic and resistance training program [69]. However, there were no changes in participant report of current pain, usual pain, or responses to quantitative sensory testing (i.e., vibration detection threshold, cold detection threshold, and hot pain threshold). The exercise prescription in this study was a supervised progressive program and started at 2 days per week of aerobic exercise (30 min at 50% VO_2_ reserve) and 1 day a week of resistance training (10 repetitions; reps) of 10 exercises targeting the abdominals, chest, back, and upper and lower extremities. Each week, the program progressed in training volume by increasing the time, intensity, or number of reps; by the end of the program, participants completed 50 min of aerobic exercise (70% VO_2_ reserve) on 2 days per week and 2 days per week of resistance training (20 reps of 10 resistance exercises). In another study, participants with painful DPN completed aerobic exercise (e.g., treadmill, stair steppers, or elliptical training) on 3 days per week and progressed from 30 min at 50% VO_2_ reserve to 50 min at 70% VO_2_ reserve [71]. Results indicated no changes in pain (i.e., average pain, worst pain, least pain, or current pain) from baseline to post-intervention; however, participants reported less pain interference following the training program, suggesting exercise reduced the amount pain interfered with their lives (e.g., mood, walking, relationships, sleep).

##### Multiple Group Studies

Two studies explored the impact of chronic exercise training on neuropathic pain using a two-arm study design [70, 72]. For example, female adults with DPN were randomly assigned to one of two 12-week interventions: (1) combined aerobic and resistance exercise training program and vitamin D supplementation or (2) vitamin D supplementation only [72]. The exercise program was progressive, and started at 20 min, and included low-intensity aerobic exercise (50% MHR) and resistance exercises (10–12 reps) at 50% of 10-repetition maximum (RM). By the end of the program, the exercise prescription consisted of 60 min of aerobic exercise at 70% MHR and the same resistance training protocol. Results indicated fewer participants in the exercise training program reported having pain (yes/no) compared to participants who completed vitamin D supplementation only. In another study, researchers conducted a randomized comparison trial to examine the influence of HIIT compared to moderate-intensity aerobic exercise [70]. Following 15 weeks of training, overweight women with DPN in the HIIT group reported significantly lower neuropathic pain scores than participants in the moderate-intensity group. There were no significant differences in neuropathic pain scores at baseline, thus this may indicate HIIT was more effective in reducing pain than moderate aerobic exercise. The HIIT program consisted of 20-min sessions on 3 days per week where participants completed a 5-min warm up followed by 8 s of vigorous-intensity sprinting (i.e., 85–95% of maximum heart rate, MHR) with 12 s of low-intensity cycling for up to 30 cycles. While HIIT training appears promising, patients with severe neuropathy and uncontrolled medical conditions were excluded from this study. Further, it took participants 2 weeks (6 sessions) to adapt to the full exercise protocol; thus, more work is needed to understand the generalizability of HIIT training.

One study used a three-arm randomized controlled trial design to examine the impact of 8 weeks of supervised combined aerobic and resistance moderate-intensity continuous training or combined HIIT on musculoskeletal and neuropathic pain compared to controls (i.e., usual care) in participants with type 2 diabetes [74]. Those in the combined aerobic and resistance continuous training intervention exercised on 4 days per week, with 2 days consisting of combined aerobic and resistance training and 2 days being aerobic exercise only. During the aerobic component, participants exercised at a moderate intensity (55–69% heart rate peak, HRpeak) for 22.5 min (combined aerobic and resistance days) or 52.5 min (aerobic only days). Resistance training was prescribed at a moderate level (i.e., fairly light to somewhat hard), lasted 30 min, and targeted the lower extremities, chest, back, shoulders, abdominals, and biceps. Participants in the combined HIIT group completed 3 sessions each week which included a 3-min aerobic exercise warm up (55–69% HRpeak), 4 min of high-intensity aerobic exercise (85–95% HRpeak), 1-min intervals of 8 high-intensity (i.e., very hard) resistance exercises with 1 min of rest between each exercise, and a 3-min aerobic exercise cool down (55–69% HRpeak). Results indicated significant reductions in musculoskeletal pain intensity from baseline to post-intervention in those who completed the combined HIIT program compared to individuals in the usual care control group. On the other hand, there were no significant changes in neuropathy symptoms or quantitative sensory testing outcomes (i.e., thermal detection thresholds, mechanical detection threshold, vibration detection threshold, and pressure pain threshold) for either exercise condition compared to controls, perhaps due to the low levels of neuropathy at baseline.

One study used a four-arm randomized controlled trial design to examine the influence of aerobic only, resistance only, or combined aerobic and resistance training programs on pain compared to controls in a sample of veterans with painful DPN [73]. In this study, participants were randomly assigned to one of four 12-week programs: aerobic exercise only, isokinetic resistance exercise only (i.e., a type of resistance training where the speed of movement is held constant throughout each rep), combined aerobic and resistance exercise, or health education. Exercise programs were held on 3 days per week while the health education program was held on 1 day per week. The aerobic exercise program progressed from 30 min (25 min at 60–70%VO_2peak_ and 5 min at 71–80% VO_2peak_) to 45 min (17 min at 60–70% VO_2peak_, 20 min at 71–80% VO_2peak_, and 8 min at 81–90% VO_2peak_) and the resistance training program progressed from 3 to 6 sets of 10 reps of isokinetic leg extensions. Those in the combined aerobic and resistance training program completed both aerobic and strength training protocols. The results indicated no significant changes in pain or sensation determined via quantitative sensory testing (i.e., vibration detection threshold, cold detection threshold, and hot pain threshold) following the exercise interventions. However, physical health-related quality of life significantly improved following the aerobic exercise only training program.

##### Clinical Considerations and Limitations

It appears the influence of exercise training on neuropathic pain in participants with type 2 diabetes is equivocal. Two of the six studies were randomized controlled trials [73, 74], and the results from these studies demonstrated improvements in quality of life following 12 weeks of moderate aerobic exercise [73] or reductions in musculoskeletal pain following 8 weeks of combined HIIT [74], but no impact on neuropathic pain. Two other studies used a randomized comparison trial design and results indicated fewer participants who completed 12 weeks of combined exercise training and vitamin D supplementation reported pain compared to those who took vitamin D supplements only [72] or that participants who completed 15 weeks of HIIT had significantly lower neuropathic pain scores compared to those in the moderate aerobic training group [70]. There were two single-arm studies; one demonstrated reduced neuropathy symptoms and “worst pain over the past month” but no changes in other measures of pain following 10 weeks of combined aerobic and resistance exercise training [69]; and the other demonstrated no changes in neuropathic pain after 16 weeks of aerobic exercise training [71]. Taken together, the evidence to date indicates limited to modest effectiveness for exercise training to reduce pain associated with neuropathic conditions.

In turn, no studies reported significant increases in neuropathic pain from baseline to post-intervention, further suggesting the potential utility of exercise training in this population; however, caution is still needed when prescribing exercise for individuals with DPN. Separate from reductions in pain, one study demonstrated reductions in pain interference [71] while another demonstrated improvement in physical health-related quality of life following moderate-intensity aerobic training [73]. Thus, exercise may be beneficial for other health outcomes besides pain in this population. However, attention to adverse events and generalizability is needed. First, adverse events were likely underreported in these studies. In the studies which included information on adverse events [69, 73, 74], no serious event was reported. However, one study reported unanticipated serious events unrelated to the study [73] and two other studies reported mild adverse events such as hyper/hypoglycemia and pain in the back, hands, knees, or legs [69, 74]. In addition, exercise studies had strict criteria for inclusion and typically excluded individuals with comorbidities and those with severe neuropathy. Further, exercise compliance is an important consideration, as the presence of neuropathy may be an added barrier to participation. For example, a recent study demonstrated significantly higher diabetic neuropathy scores in patients who dropped out of an exercise training trial compared to those who completed the study [74].

#### Other Neuropathies: CIPN and HIV-Related Distal Symmetric Polyneuropathy

Three studies examined the influence of neuropathic pain resulting from conditions other than DPN (i.e., CIPN, HIV-related distal symmetric polyneuropathy) [75–77]. Two studies examined the influence of combined exercise training on pain in participants with CIPN [75, 76], and one study examined the influence of either aerobic exercise only or resistance exercise only in participants with HIV-related distal symmetric polyneuropathy [77].

##### CIPN

Two studies examined the impact of home-based combined exercise training on neuropathic pain in participants with CIPN [75, 76]. For example, in a randomized controlled trial, participants with CIPN undergoing treatment reported lower neuropathic pain scores following 10 weeks of home-based balance and strength exercises compared to individuals in the usual care group [76]. Researchers prescribed 30 min per day (7 days per week) of strength and balance exercises which consisted of exercises lying down for 7 min (ankle motion, hip abduction, and straight leg raises), sitting exercises for 13 min (digit movements, wrist motion, elbow flexion/extension, knee flexion extension, and toe tapping), and 10 min of standing balance exercises (one legged stance, toe stands, hip extensions, and tandem forward walking). In a single-arm trial, breast cancer survivors with CIPN underwent a 10-week moderate-intensity walking and resistance exercise program [75] to examine whether exercise training influenced CIPN symptoms (including pain characteristics) and quality of life (i.e., how troublesome their symptoms were). Participants were asked to complete home-based progressive exercise which consisted of walking at 55–65% MHR starting at 2 days per week for 10 min and ending with 30 min per day for 5 days per week. In addition, the exercise program consisted of resistance exercise on 3 days per week using resistance bands. By the end of the study, participants reported their symptoms were less troublesome and fewer individuals reported experiencing pain symptoms such as unpleasant sensations of the skin, abnormal sensitivity to touch, and sudden bursts of pain.

##### HIV-Related Distal Symmetric Polyneuropathy

One study examined the impact of exercise on HIV patients with distal symmetric polyneuropathy [77]. In this randomized controlled trial, participants engaged in 12 weeks of supervised moderate progressive aerobic exercise, or moderate progressive resistance exercise, or an HIV education control on 3 days per week for 30 min per day. The aerobic exercise program included stationary biking in three phases: warm-up (5 min with no resistance), aerobic (20 min with low-moderate resistance), and cool-down (5 min with no resistance). The aerobic phase progressed from 40% MHR for the first 6 weeks to 65% MHR for the last 6 weeks. The resistance exercise program consisted of three phases: warm-up (5 min stretching), resistance exercise (20 min exercises), and cool-down (5 min stretching). The resistance exercise protocol consisted of 2 sets of 10 reps of resistance exercises targeting the lower extremities (i.e., quadriceps, hamstrings, tibialis anterior, and gastrocnemius) at 40% 1-RM for the first 6 weeks and progressed to 2 sets of 10 reps of the same exercises at 65% 1-RM max for the remaining 6 weeks. Participants in both exercise groups reported reduced “pain over the last 24 h” following the intervention, whereas the control group reported no significant change in pain.

##### Clinical Considerations and Limitations

Preliminary evidence examining the utility of exercise training in reducing neuropathic pain in CIPN or HIV-related distal symmetric polyneuropathy is generally supportive, although there are important considerations. Two randomized controlled trials demonstrated exercise training reduced neuropathic pain following 10 weeks of combined balance and strength training [76] or following 12 weeks of either moderate aerobic exercise training or 12 weeks of moderate resistance exercise training [77]. As more evidence accumulates, future researchers should consider potential recruitment barriers. For example, patients were recruited through clinics or medical registries and out of those identified, only 28–67% were enrolled, in part due to stringent inclusion/exclusion criteria [76, 77] and/or an unwillingness to participate [75, 77]. Similarly, exercise compliance is an important consideration as there was low retention (42%) in one study yet high retention (88–96%) in two other studies. The studies with high retention were conducted in a clinical setting (day care unit of a tertiary care hospital [76]; rehabilitation clinics [77]), whereas the study with low retention was conducted outside of the clinical setting [75]. Thus, study setting may be an important factor for exercise compliance. Further work is needed to understand factors that contribute to enrollment, adherence, and retention of participants to exercise programs.

#### Summary

While the clinical literature base suggests exercise may be beneficial in populations with neuropathic pain, not all studies support this claim and there are several limitations that need to be understood when discussing the effect of exercise on neuropathic pain. There is evidence that exercise training reduced worst pain over the last month [69], pain over the past 24 h [77], pain or neuropathy scores [69, 70, 75, 76], the presence of pain [72], musculoskeletal pain [74], and pain interference [71] and improved quality of life [73]. At the same time, there is evidence that exercise training had no impact on neuropathic pain [73, 74] or that it only influenced one aspect of pain while others remained unchanged [69]. Further, randomized controlled trials were rare and it is therefore unclear if effects of the exercise intervention are due to exercise or other factors such as the attention given to participants or the natural course of time; thus, conclusions described in this summary are preliminary. Further, several studies included small sample sizes which may reflect the difficulty in recruiting patients with neuropathy to participate in exercise training. Similarly, stringent inclusion/exclusion criteria make it difficult to generalize to the population with neuropathic pain at large. Further, more work is needed to understand compliance to exercise programs in neuropathic pain populations. One factor may be transient increases in pain or other potential adverse events that may occur during exercise training [69, 74]. These events are likely underreported in the current literature and the literature base would benefit from reporting adverse events that can accompany exercise training, including, as suggested by Cox and associated (2020), rheumatological and musculoskeletal symptoms [78]. In general, there is a need for more systematic and rigorous investigations that include consistent pain measures to better compare across studies, larger sample sizes, control groups to account for common confounding variables, and comparison studies to help provide specific exercise prescriptions. Further, little is known about the mechanisms behind exercises’ effects on neuropathic pain in the clinical literature. An understanding of these mechanisms may shed light on the potential for exercise as a treatment option for neuropathic pain.

## Mechanisms

The majority of mechanistic insight comes from preclinical data where specific mechanisms were measured in conjunction with nociceptive behavioral responses using different exercise programs. Preclinical evidence suggests that exercise may reduce neuropathic pain through normalizing microglia activation, balancing pro- and anti-inflammatory responses, upregulating heat shock protein 72, and producing alterations in neurotransmitter and neuromodulatory systems (see below).

### Microglia

Activation of microglia in response to neuronal damage may stimulate a cascade of events leading to increased nociceptive neuronal excitability and ultimately the experience of neuropathic pain [34, 79]. Recent preclinical evidence demonstrated normalization of microglial activity [17, 21, 28, 44], as well a shift towards a healthy balance of inflammatory markers [32, 43] in the DRG in response to exercise training. Thus, exercise may promote pain reductions through normalizing microglial activity in the DRG. However, these changes often occur in conjunction with alterations in inflammatory markers.

### Inflammation

Pro-inflammatory markers such as tumor necrosis factor-α, interleukin-1β, and interleukin-6 have been studied in the context of both neuropathic pain development and exercise. It is thought that markers such as these are released in response to microglial activation after nerve damage [79]. Interestingly, pro-inflammatory markers are elevated in response to acute exercise, however chronic exercise training stimulates an anti-inflammatory response [80]. Therefore, it is possible exercise influences pain through inflammatory marker change [81]. In fact, most preclinical evidence demonstrates an increase in pro-inflammatory markers following nerve injury and an attenuation of this increase after exercise [18, 26, 30–32, 43, 45, 52, 82]. Some studies demonstrate an increase [26, 31, 43] or a normalization [31] of interleukin-10, an anti-inflammatory cytokine, levels in response to exercise. Recent work implicated the role of interleukin-4 in this mechanism, as interleukin-4 antagonism or knockout mice reversed the analgesic effect of exercise training [32]. Therefore, exercise may lead to decreases in neuropathic pain by preventing pro-inflammatory cytokine upregulation and promoting anti-inflammatory cytokine release.

### Heat Shock Protein 72

Heat shock protein 72 is a protein expressed in many organs, typically in response to stressful environments, with the primary function of aiding other proteins in healthy development [83]. Recent work demonstrated higher levels of heat shock protein 72 and lower levels of hyperalgesia (thermal, mechanical) and allodynia (mechanical) in exercised mice compared with non-exercised mice [19, 49]. Thus, demonstrating the potential role of Hsp72 as a mechanism for reduced hyperalgesia and allodynia following exercise training.

### Neurotrophins

Exercise may facilitate healthy nerve growth in damaged pain sensory pathways through influencing neurotrophic proteins (e.g., brain derived neurotrophic factor). Upregulation of brain derived neurotrophic factor, stemming from microglial activation in the DRG, is thought to impair nociceptive processing, producing hyperalgesia and allodynia [72]. In SNI mice, exercise appeared to ameliorate these increases [21, 24, 32, 34, 35, 46, 84] leading to decreased hyperalgesia. Other neurotrophins have been shown to normalize in response to exercise as well [37, 84]. Some studies concluded exercise may prevent pathological nociceptive neuronal growth [54, 81] and promotion of healthy nerve regeneration in the nociceptive pathway [17, 46, 69].

### Neurotransmitters (Dopamine, Epinephrine, Gamma-aminobutyric Acid, Serotonin)

There is evidence to support the involvement of neurotransmitters in attenuating neuropathic nociception following exercise training [22, 27, 29, 33]. Exercise-induced reductions in allodynia and hyperalgesia were prevented by inhibition of the dopaminergic pathway [29]. This suggests decreases in pain after chronic exercise in neuropathic pain may involve the dopaminergic system. At the same time, the serotonergic system was implicated after researchers documented increased levels of serotonin, upregulation of serotonergic receptors, and reduced serotonin transporters in the brainstem [22, 33]. In addition, exercise increased levels of adrenergic receptors (alpha and beta) found after nerve injury [33]. Further, recent research suggested gamma-aminobutyric acid (GABA) may play a mechanistic role, due to normalization of GABA in the nociceptive pathway in response to exercise [27]. Perhaps exercise training improves neurotransmitter tone in the dorsal horn.

### Endogenous Opioids

Exercise effects in animal models of neuropathic pain may be mediated by changes in endogenous opioids. For example, exercise training reduced thermal and tactile hypersensitivity and increased opioid content in the rostral ventromedial medulla and the midbrain periaqueductal gray area in spinal nerve-ligated animals. However, exercise effects were reversed by administration of opioid receptor antagonists [36]. In animals with SNI, the effects of exercise-induced reductions in mechanical hypersensitivity were reversed by administration of naloxone (an opioid receptor antagonist) [34]. Also, exercise-induced analgesia in diabetic rats was reversed in a dose-dependent manner with the administration of naloxone [48]. Further, swimming exercise reduced mechanical allodynia in an animal model of CRPS-1 but the anti-allodynic effect of exercise was reversed by pretreatment of naloxone [53].

### Endocannabinoids

Pharmacological manipulation of the endocannabinoid system reduces nociceptive behaviors such as mechanical allodynia, cold allodynia, mechanical hyperalgesia, or heat hyperalgesia in preclinical models of SNI (e.g., [85–88]), SCI (e.g., [89–91]), CIPN (e.g., [92–98]), and DPN (e.g., [99–102]). While no research to date has examined the endocannabinoid system as a mechanism for exercise-induced reductions in neuropathic pain, the endocannabinoid system has been implicated in exercise-induced hypoalgesia (i.e., acute pain reductions following exercise) in healthy humans performing isometric exercise [103]; thus, future research is warranted.

## Conclusions

The previous sections summarized evidence examining the efficacy of exercise training to reduce neuropathic pain. Preclinical evidence demonstrated treadmill running protocols consisting of 30–60 min of exercise performed 3–5 days/week for 1–15 weeks were effective in reducing neuropathic nociception. Swimming protocols were also effective and consisted of 30–60 min/day of swimming performed 5–7 days/week for 4–10 weeks. Clinical evidence demonstrated 10–12 weeks of moderate-intensity combination training (i.e., aerobic and resistance exercise) or 15 weeks of HIIT reduced sensory (i.e., pain intensity, pain characteristics) or affective (i.e., how troublesome the pain is) components of neuropathic pain. One program consisting of 16 weeks of moderate-intensity aerobic exercise led to reductions in pain interference but not pain intensity [71]. Taken together, the preclinical and clinical evidence suggests further study is warranted. Randomized controlled trials are needed to further translate the preclinical evidence base to the clinical realm. Specifically, systematic investigations into the mode, intensity, frequency, and duration of exercise that reduce neuropathic pain are needed. In addition, consistent patient-reported and clinical outcomes as well as potential mechanisms should be included in future studies. As a result, the literature can move towards a consensus and practitioners may begin to provide specific exercise recommendations for patients experiencing neuropathic pain.

## Data Availability

Not applicable

## Abbreviations

CIPN: Chemotherapy-induced peripheral neuropathy
CRPS: Complex regional pain syndrome
DPN: Diabetic peripheral neuropathy
DRG: Dorsal root ganglion
GABA: Gamma-aminobutyric acid
HIIT: High-intensity interval training
HIV: Human immunodeficiency virus
HR_peak_: Peak heart rate
MHR: Maximum heart rate
SCI: Spinal cord injury
SNI: Sciatic nerve injury
VO_2_: Oxygen uptake
